# Anterior cingulate cortex is crucial for contra- but not ipsi-lateral electro-acupuncture in the formalin-induced inflammatory pain model of rats

**DOI:** 10.1186/1744-8069-7-61

**Published:** 2011-08-22

**Authors:** Ming Yi, Haolin Zhang, Lixing Lao, Guo-Gang Xing, You Wan

**Affiliations:** 1 Neuroscience Research Institute, Peking University, 38 Xueyuan Road, Beijing 100191, China; 2 Center for Integrative Medicine, School of Medicine, University of Maryland, 685 W. Baltimore Street, MSTF Rm 8-22, Baltimore, MD, 21201, USA; 3 Key Laboratory for Neuroscience, Ministry of Education/Ministry of Public Health, 38 Xueyuan Road, Beijing 100191, China

**Keywords:** pain, acupuncture, electroacupuncture, anterior cingulate cortex, formalin test

## Abstract

Acupuncture and electro-acupuncture (EA) are now widely used to treat disorders like pain. We and others have shown previously that current frequency, intensity and treatment duration all significantly influence the anti-nociceptive effects of EA. There is evidence that stimulating sites also affect the antinociception, with EA applied ipsilaterally to the pain site being more effective under some pain states but contralateral EA under others. It was recently reported that local adenosine A1 receptors were responsible for ipsilateral acupuncture, but what mechanisms specifically mediate the anti-nociceptive effects of contralateral acupuncture or EA remains unclear. In the present study, we applied 100 Hz EA on the ipsi- or contra-lateral side of rats with inflammatory pain induced by intra-plantar injection of formalin, and reported distinct anti-nociceptive effects and mechanisms between them. Both ipsi- and contra-lateral EA reduced the paw lifting time in the second phase of the formalin test and attenuated formalin-induced conditioned place aversion. Contralateral EA had an additional effect of reducing paw licking time, suggesting a supraspinal mechanism. Lesions of rostral anterior cingulate cortex (ACC) completely abolished the anti-nociceptive effects of contra- but not ipsi-lateral EA. These findings were not lateralized effects, since injection of formalin into the left or right hind paws produced similar results. Overall, these results demonstrated distinct anti-nociceptive effects and mechanisms between different stimulating sides and implied the necessity of finding the best stimulating protocols for different pain states.

## Findings

Acupuncture, applied by inserting long needles into specific points in the body (acupoints), has been used in China for over two thousand years to treat a variety of diseases including pain. Electro-acupuncture (EA), manipulated by passing electric currents through acupuncture needles, is also widely used and allows a more objective control over stimulating parameters. EA activates/deactivates a variety of brain regions and promotes the release of endogenous opioid peptides, which are responsible for mediating its analgesic effects [1,2]. Work from our laboratory and others shows that stimulating frequency, intensity and duration all significantly influence the analgesic effects of EA and may have different mechanisms [1-5]. For example, 2 Hz EA accelerates the release of enkephalin, β-endorphin and endomorphin, whereas 100 Hz EA selectively increases the release of dynorphin [1]. Stimulating sites also affect the antinociception, e.g., ipsilateral EA (stimulating at locations ipsilateral and close to pain sites) versus contralateral EA (stimulating at locations contralateral to pain sites). Goldman et al. [6] reported significant analgesic effects of local (ipsilateral) but not distal (contralateral) acupoints in a mouse model of inflammatory pain. Somers and Clemente obtained opposite results that transcutaneous electric nerve stimulation on the contra- but not ipsi-lateral side of neuropathic pain showed anti-nociceptive effects in rats [7]. These findings are consistent with traditional acupuncture theories which suggest that different acupoints should be stimulated for various pain states, depending on their location, intensity, modality, and duration, to achieve the greatest therapeutic effects [8]. A recent study showed that the anti-nociceptive effects of ipsilateral (local) acupuncture are mediated by local adenosine A1 receptors [6], but what mechanisms mediate contralateral (distal) acupuncture or EA remains unclear.

**Figure 1:**
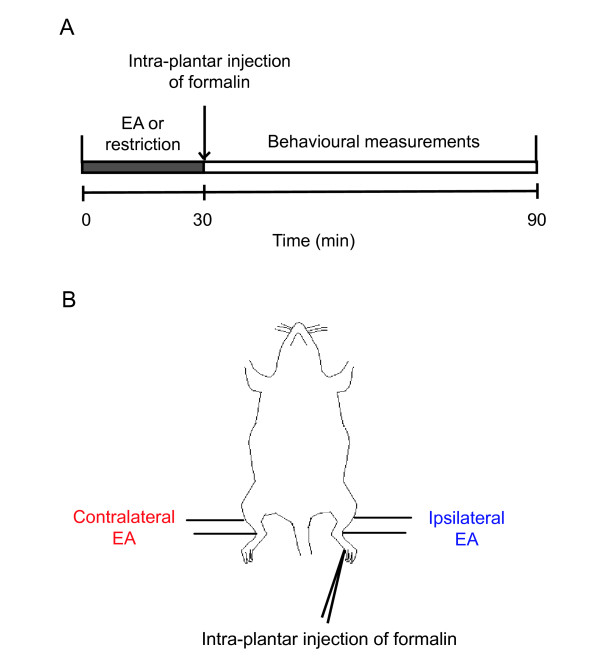
**Schematic diagrams of the formalin model of inflammatory pain and EA stimulation**. (A) Rats received EA or restriction alone before intra-plantar injection of formalin. (B) The rat received formalin injection in the left hind paw. Ipsi- or contra-lateral EA was applied to "Zusanli" and "Sanyinjiao" in the left or right leg of the rat respectively. In the last part of the study, formalin was injected to the opposite (right) paw and ipsi- or contra-lateral EA referred to EA in the right or left leg, respectively.

**Figure 2:**
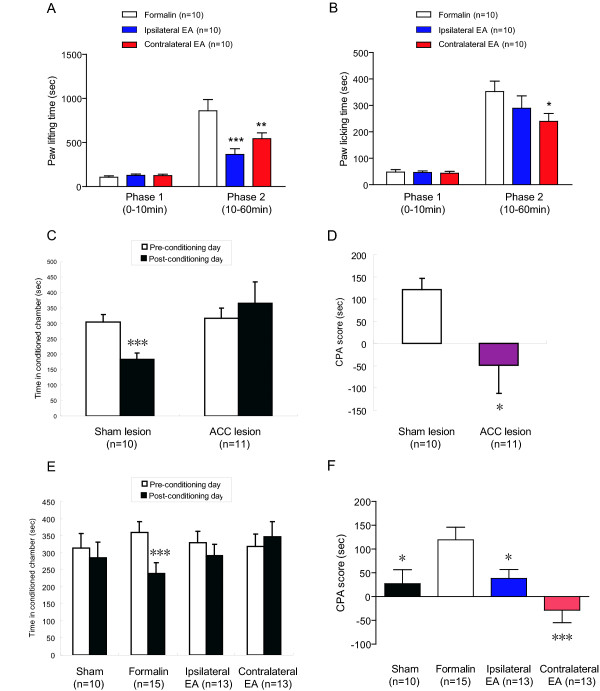
**Effects of ipsilateral or contralateral EA on pain sensation and affection in the formalin model**. ***A***, Either ipsilateral or contralateral EA significantly decreased the paw lifting time in the second phase of the formalin test. ***B***, Contralateral but not ipsilateral EA reduced paw licking time in the second phase. ***C***, ACC lesions reversed F-CPA, which was a significant decrease of the time spent in the formalin-paired compartment. ***D***, ACC lesions significantly decreased CPA scores. ***E***, F-CPA was reversed by either ipsilateral or contralateral EA. ***F***, Either ipsilateral or contralateral EA significantly decreased CPA scores. * p < 0.05; ** p < 0.01; *** p < 0.005 compared with the formalin group (A, B, D and F) and between pre-conditioning and post-conditioning days (C and E). All data were presented as means ± SEM.

**Figure 3:**
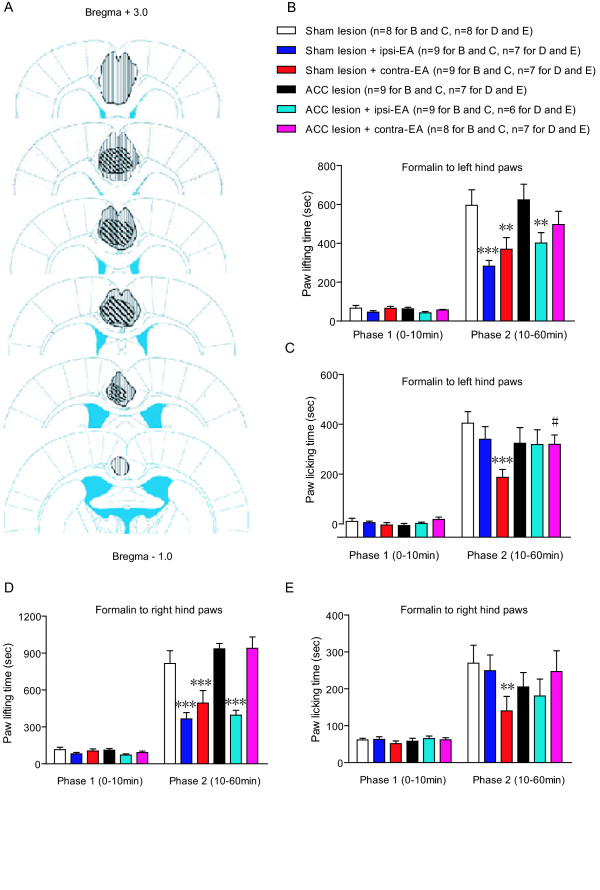
**Effects of rostral ACC lesions on ipsilateral or contralateral EA in the formalin model**. ***A***, Schematic representation of the maximum (longitudinal line) and minimum (diagonal line) extent of bilateral ACC lesions. ***B ***and ***D***, With formalin injected to the left (B) or right (D) hind paws, rostral ACC lesions reversed the decrease of paw lifting time by contralateral EA, but that by ipsilateral EA was still present. ***C ***and ***E***, With formalin injected to the left (C) or right (E) paws, bilateral rostral ACC lesions abolished the decrease of paw licking time by contralateral EA. * p < 0.05; ** p < 0.01; *** p < 0.001, compared with the formalin group of the corresponding lesion type (sham lesion or ACC lesion). # p < 0.05, compared with the formalin + contralateral EA group of sham lesions. All data were presented as means ± SEM.

To answer this question, we first established the classic acute inflammatory pain model of rats by intra-plantar injection of formalin, and applied 100 Hz EA on the ipsi- or contra-lateral side of the injected hind paw (Figure 1). Two nociceptive responses, paw licking and paw lifting, were monitored and used as parameters of pain intensity [9]. Interestingly, either ipsi- or contra-lateral EA significantly decreased nociceptive behaviours in the second phase of the test, but via different mechanisms. Either ipsi- or contra-lateral EA reduced the paw lifting time (Figure 2A. Formalin group 859.0 ± 128.4 s; ipsilateral EA group 364.7 ± 64.4 s, p < 0.001 compared with the control group; contralateral EA group 542.8 ± 66.5 s, p < 0.01), only the latter reduced the paw licking time (Figure 2B. Formalin group 352.3 ± 39.4 s; ipsilateral EA group 289.5 ± 46.7 s, p > 0.05; contralateral EA group 239.7 ± 30.0 s, p < 0.05). Nociceptive behaviours in the first phase were not different across groups (Figure 2A and 2B. Paw lifting time: formalin group 105.5 ± 16.4 s; ipsilateral EA group 127.2 ± 15.3 s; contralateral EA group 124.2 ± 16.6 s. Paw licking time: formalin group 47.7 ± 9.7 s; ipsilateral EA group 46.4 ± 5.8 s; contralateral EA group 43.6 ± 6.8 s. p > 0.05 for all comparisons). These findings were further confirmed in our lesion experiments described later (Figure 3B-E).

Pervious studies have established dissociation between the sensory-discriminative and the emotional-affective dimensions of pain [10,11]. Lesioning the anterior cingulate cortex (ACC) does not change pain sensation but significantly relieve pain affection, indicated from the formalin-conditioned place aversion (F-CPA) task [11] (Figure 2C and 2D). Time spent in the pain-paired compartment decreased from 304.9 ± 23.5 s on the pre-conditioning day to 183.7 ± 19.6 s on the post-conditioning day (p < 0.001) in the sham lesion group and remained unchanged in the ACC lesion group (315.8 ± 33.1 s on the pre-conditioning day and 364.9 ± 68.6 s on the post-conditioning day, p > 0.05). The CPA score was 121.2 ± 25.1 in the sham lesion group and - 49.1 ± 63.2 in the ACC lesion group (p < 0.05). EA relieves pain sensations in a variety of pain states [1-9], but whether it can attenuate pain affection has never been tested. We trained a group of experimentally naive rats in the F-CPA task as previously described [11]. Formalin injection induced a clear CPA (Sham group injected with normal saline: 313.1 ± 43.9 s on the pre-conditioning day and 285.9 ± 45.5 s on the post-conditioning day, p > 0.05. Formalin group: 359.1 ± 31.5 s on the pre-conditioning day and 239.6 ± 30.6 s on the post-conditioning day, p < 0.001), which was inhibited by either ipsi- or contra-lateral EA (ipsilateral EA group: 330.2 ± 32.1 s on the pre-conditioning day and 292.0 ± 33.6 s on the post-conditioning day, p > 0.05. Contralateral EA group: 318.1 ± 37.3 s on the pre-conditioning day and 346.7 ± 45.0 s on the post-conditioning day, p > 0.05) (Figure 2E). A similar conclusion could be drawn when CPA scores were calculated (Figure 2F. Formalin group: 119.5 ± 26.3; sham group 27.2 ± 29.4, p < 0.05; ipsilateral EA group 38.2 ± 18.5, p < 0.05; and contralateral EA group - 28.6 ± 26.3, p < 0.001). Contralateral EA had a better but insignificant effect than ipsilateral EA, which paralleled the fact that contra- but not ipsi-lateral EA reduced pain licking time (Figure 2B). Thus, both ipsi- and contra-lateral EA attenuated pain affection in the formalin model.

The paw licking behaviour in the formalin model parallels neural activities in the rat forebrain [12], which leads to the suggestion that paw lifting behaviours following chemical stimulant injection represent the outcome of peripheral or spinal mechanisms related with the sensory component of pain, whereas paw licking behaviours represent supraspinal mechanisms involving negative hedonic value of the painful stimulus [9,12]. In this point of view, the mechanisms between ipsi- and contra-lateral EA might be different: ipsi-lateral EA exerts its anti-nociceptive effects mainly through peripheral or spinal mechanisms, as previously shown [6], whereas contra-lateral EA involves an additional supraspinal component.

We tested whether lesions of rostral ACC, a forebrain region crucial for pain affection [10,11], would affect the anti-nociceptive effects of ipsi- or contra-lateral EA. Our focus on this region stemmed from several sources. Firstly, ACC is part of the medial pain pathway and has strong connections with a variety of brain regions crucial for pain modulation, including the medial prefrontal cortex, hippocampus, amygdala, orbitofrontal cortex, periaqueductal grey and autonomic brainstem motor nuclei [13]. Secondly, neuroimaging studies repeatedly report that both acupuncture and pain modulate the limbic-paralimbic-neocortical network including ACC [14,15]. Thirdly, EA relieves pain-related negative emotions (Figure 2E and 2F), where ACC has a significant role [10,11]. Finally, a multi-unit recording study performed on rats reported a decrease of nociceptive responses in the ACC after peripheral electrical stimulation [16].

Bilateral rostral ACC lesions (Figure 3A) induced a mild decrease of paw licking behaviours after intra-plantar formalin injection, as previously reported [9,10]. After ACC lesions, the anti-nociceptive effects of contra-lateral EA were completely abolished, since neither paw lifting nor paw licking time showed significant differences from the control group (Figure 3B and 3C, Table 1). In contrast, the decrease of paw lifting time by ipsilateral EA was still present (Figure 3B, Table 1).

**Table 1:** Effects of ACC lesions on the anti-nociceptive effects of EA in rats with formalin injection

	Paw lifting time (s)		Paw licking time (s)	
	
	Phase 1	Phase 2	Phase 1	Phase 2
**Formalin to left paws**				
Sham lesion	66.3 ± 13.9	595.5 ± 80.0	61.3 ± 12.2	440.1 ± 44.7
Sham lesion + ipsi-EA	45.8 ± 8.0	282.7 ± 28.7 ***	56.1 ± 6.3	378.3 ± 48.2
Sham lesion + contra-EA	65.3 ± 10.5	369.7 ± 59.8 **	47.0 ± 9.1	231.3 ± 30.6 ***
ACC lesion	63.1 ± 8.1	623.6 ± 80.9	45.0 ± 8.2	362.6 ± 60.9
ACC lesion + ipsi-EA	42.3 ± 6.4	401.7 ± 53.1 **	53.0 ± 5.4	357.9 ± 56.8
ACC lesion + contra-EA	57.3 ± 3.2	496.9 ± 68.2	68.5 ± 9.2	358.8 ± 36.0^#^
**Formalin to right paws**				
Sham lesion	115.6 ± 19.2	815.5 ± 104.4	61.5 ± 4.7	269.6 ± 48.4
Sham lesion + ipsi-EA	81.0 ± 11.9	365.1 ± 50.9 ***	63.0 ± 7.2	249.1 ± 42.7
Sham lesion + contra-EA	104.7 ± 16.4	494.0 ± 101.1 ***	51.4 ± 7.5	140.1 ± 39.2 **
ACC lesion	107.7 ± 11.5	854.1 ± 88.1	58.1 ± 7.9	205.3 ± 39.0
ACC lesion + ipsi-EA	71.0 ± 10.5	395.9 ± 39.2 ***	65.1 ± 7.1	180.7 ± 45.9
ACC lesion + contra-EA	91.3 ± 12.6	939.2 ± 92.6	61.7 ± 5.9	246.7 ± 56.3

* p < 0.05; ** p < 0.01; *** p < 0.001, compared with the formalin group of the corresponding lesion type (sham lesion or ACC lesion). # p < 0.05, compared with the formalin + contralateral EA group of sham lesions.

All the above experiments were performed with formalin injection in left hind paws. To exclude the possibility that these findings were lateralized to one side, we repeated the lesion experiment in a new cohort of rats with formalin injection in their right hind paws and obtained similar results (Figure 3D and 3E). Thus, an intact rostral ACC was indispensable for contralateral EA but not ipsilateral EA. Other mechanisms, such as those in the periphery [6] and in the descending pathways [1,2], might mediate the antinociception of ipsilateral EA.

How ACC mediates the anti-nociceptive effects of contralateral EA requires further investigation but several potential mechanisms are proposed here. Nociceptive neurons in ACC have large bilateral receptive fields that often include the whole body [17,18], which implies that contralateral EA may exert its anti-nociceptive effects by a competitive mechanism: nociceptive signals from one side of the body may be inhibited by EA signals from the contralateral side. Anatomically, ACC is interconnected with several regions involved in pain modulation, such as amygdala and periaqueductal grey [13]. At a short-term level, ACC activation most likely triggers the descending inhibitory system, whose lesions abolish acupuncture analgesia [2]. In the long-term run, ACC activation may activate/deactivate affection-related regions to induce an inhibition of emotional responses that is observed in the F-CPA task.

Additionally, the mechanisms of the formalin test's two phases are different: the first phase results from the activation of peripheral nociceptors whereas the second phase reflects the development of inflammation and central sensitization. In our study, EA only attenuated nociceptive behaviours in the second phase of the test, suggesting a potential mechanism of desensitization, which has been reported in the spinal dorsal horn [19]. A similar mechanism may be expected for ACC, which has a high level of neuroplasticity essential for the formation of chronic pain and pain-related emotional changes [20,21]. It has been shown that prolonged peripheral stimulation induced expression of the immediate early gene c-fos bilaterally in the ACC [22] and changed its neuronal responses to subsequent peripheral stimuli [23].

Overall, the present study indicated distinct anti-nociceptive effects and mechanisms between ipsi- and contra-lateral EA. ACC is crucial for the latter but not the former type of EA antinociception. These data support the traditional acupuncture theory that different acupoints should be selected for different pain states [8]. However, further dedicated investigation on this issue is required, to obtain acupunctural protocols with greatest therapeutic effects for different pain states.

## Methods

### Subjects

Adult Sprague-Dawley rats weighing 160-220 g at the beginning of the experiment were provided by the Department of Experimental Animal Sciences, Peking University Health Science Center. All animal experimental procedures were conducted in accordance with the guidelines of the International Association for the Study of Pain and were approved by the Animal Care and Use Committee of the University. The behavioural experimenters were kept blind from the groupings of the rats.

### The formalin model of acute inflammatory pain and EA stimulation

All subjects were divided into three groups: the formalin group, which received formalin injection in their left hind paws without EA treatment, and the ipsi- and contra-lateral EA groups, which received EA application on their left and right leg before formalin injection, respectively (Figure 1). For EA application, two commonly used acupoints, "Zusanli" (ST 36, 4 mm lateral to the anterior tubercle of the tibia, which is marked by a notch) and "Sanyinjiao" (SP 6, 3 mm proximal to the medial melleolus, at the posterior border of the tibia) [22], were stimulated with square waves of 0.2 ms in pulse width and 100 Hz in frequency from a Han's Acupoint Nerve Stimulator (HANS, LH series, manufactured in our university). Their intensities were increased in a stepwise manner at 1.0-1.5-2.0 mA, each lasting for 10 min [22].

Immediately after EA application or restriction, each rat was administered a 0.1 ml injection of 8% formalin solution into the plantar surface of the left hind paw. After injection, the rat was placed into a 30 × 30 × 30 cm Plexiglas chamber. Behavioural testing immediately began and lasted for 60 min. The amount of time the animal spent with the injected paw lifted or licked was recorded, respectively [11] for the two phases after formalin injection (0 - 10 min for phase 1, and 10 - 60 min for phase 2). These data were analyzed with two-way ANOVA followed by LSD post hoc tests.

### Formalin-conditioned place aversion (F-CPA)

Animals were trained in a black-walled shuttle box (72 × 25 × 30 cm, length × width × height) consisted of three interconnected compartments (left, middle and right) with distinctive visual and tactile cues. A consecutive 4-day training procedure was used. On day 1 (pre-conditioning day), each rat was allowed free access to all three compartments for 15 min while the time spent in each compartment was recorded to assess its innate place preference. On day 2, rats were randomly restrained in the left or right compartments for 45 min. On day 3, rats received no treatment (sham group), or an injection of formalin (8%, 0.1 ml) into the left hind paw (formalin, ipsi- and contra-lateral EA groups) and were then counterbalanced with 45-min restraint in the opposite compartment (right or left, respectively). Rats in the ipsi- or contra-lateral EA group received EA treatment in their left or right leg before formalin injection as described above. On day 4 (post-conditioning day), each rat was again allowed free access to all three compartments for 15 min, and the time spent in each compartment was recorded. CPA scores represented the time spent in the treatment-paired compartment on the pre-conditioning day (day 1) minus the time spent in the same conditioning compartment on the post-conditioning day (day 4) [13]. Time in pre- and post-conditioning days in each group was compared with paired t test and CPA scores were compared with one-way ANOVA with Newman-Keuls posttest.

### ACC lesions and histology

Bilateral electrolytic lesions of rostral ACC were performed with a constant current of 1 mA for 20 s to the following stereotaxic coordinates: AP +1.6 mm from bregma, ML ± 0.6 mm, DV 2.0 mm. Animals of sham lesions underwent the same surgical procedure except for passing the current. One week after the surgery, the animals received a left intra-plantar injection of 0.1 ml 10% formalin solution. Six groups were included: sham lesion, sham lesion + ipsilateral EA, sham lesion + contralateral EA, ACC lesion, ACC lesion + ipsilateral EA, and ACC lesion + contralateral EA. Formalin was injected in left or right hind paw in two independent cohorts of animals. EA was applied as described earlier. Histological confirmation of ACC lesions was performed by microscopic inspection of 50 μm coronal brain sections with Nissl staining.

## List of abbreviations used

ACC: anterior cingulate cortex; EA: electro-acupuncture; F-CPA: formalin-conditioned place aversion.

## Competing interests

The authors declare that they have no competing interests.

## Authors' contributions

MY and ZH contributed equally for this manuscript. MY, LL, GGX and YW raised the hypothesis. MY, HZ, LL, GGX and YW designed the experiment. MY and HZ carried out the experiments and performed the data analysis. MY, HZ and YW drafted the manuscript. All authors read and approved the final manuscript.

## References

[B1] Han JS Acupuncture: neuropeptide release produced by electrical stimulation of different frequencies Trends Neurosci 2003 26 17 22 10.1016/S0166-2236(02)00006-1 12495858

[B2] Zhao ZQ Neural mechanism underlying acupuncture analgesia Prog Neurobiol 2008 85 355 375 10.1016/j.pneurobio.2008.05.004 18582529

[B3] Schimek F Chapman CR Gerlach R Colpitts YH Varying electrical acupuncture stimulation intensity: effects on dental pain-evoked potentials Anesth Analg 1982 61 499 503 6979271

[B4] Taguchi T Taguchi R Effect of varying frequency and duration of electroacupuncture stimulation on carrageenan-induced hyperalgesia Acupunct Med 2007 25 80 86 10.1136/aim.25.3.80 17906601

[B5] Wang SM Lin EC Maranets I Kain ZN The impact of asynchronous electroacupuncture stimulation duration on cold thermal pain threshold Anesth Analg 2009 109 932 935 10.1213/ane.0b013e3181ad9292 19690269

[B6] Goldman N Chen M Fujita T Xu Q Peng W Liu W Jensen TK Pei Y Wang F Han X Chen JF Schnermann J Takano T Bekar L Tieu K Nedergaard M Adenosine A1 receptors mediate local anti-nociceptive effects of acupuncture Nat Neurosci 2010 13 883 888 10.1038/nn.2562 20512135 PMC3467968

[B7] Somers DL Clemente FR Transcutaneous electrical nerve stimulation for the management of neuropathic pain: the effects of frequency and electrode position on prevention of allodynia in a rat model of complex regional pain syndrome type II Phys Ther 2006 86 698 709 16649893

[B8] Unschuld PU Huang Di Nei Jing Su Wen 2003 University of California Press, Berkeley

[B9] Donahue RR LaGraize SC Fuchs PN Electrolytic lesion of the anterior cingulate cortex decreases inflammatory, but not neuropathic nociceptive behaviour in rats Brain Res 2001 897 131 138 10.1016/S0006-8993(01)02103-5 11282366

[B10] Rainville P Duncan GH Price DD Carrier B Bushnell MC Pain affect encoded in human anterior cingulate but not somatosensory cortex Science 1997 277 968 971 10.1126/science.277.5328.968 9252330

[B11] Johansen JP Fields HL Manning BH The affective component of pain in rodents: direct evidence for a contribution of the anterior cingulate cortex Proc Natl Acad Sci USA 2001 98 8077 8082 10.1073/pnas.141218998 11416168 PMC35470

[B12] Porro CA Cavazzuti M Lui F Giuliani D Pellegrini M Baraldi P Independent time courses of suprasinal nociceptive activity and spinally mediated behaviour during tonic pain Pain 2003 104 291 301 10.1016/S0304-3959(03)00015-0 12855340

[B13] Wyss JM Sripanidkulchai K The topography of the mesencephalic and pontine projections from the cingulate cortex of the rat Brain Res 1984 293 1 15 10.1016/0006-8993(84)91448-3 6704709

[B14] Wu MT Sheen JM Chuang KH Yang P Chin SL Tsai CY Chen CJ Liao JR Lai PH Chu KA Pan HB Yang CF Neuronal specificity of acupuncture response: an fMRI study with electroacupuncture Neuroimage 2002 16 1028 1037 10.1006/nimg.2002.1145 12202090

[B15] Hui KK Liu J Makris N Gollub RL Chen AJ Moore CI Kennedy DN Rosen BR Kwong KK Acupuncture modulates the limbic system and subcortical gray structures of the human brain: evidence from fMRI studies in normal subjects Hum Brain Mapp 2000 9 13 25 10.1002/(SICI)1097-0193(2000)9:1<13::AID-HBM2>3.0.CO;2-F 10643726 PMC6871878

[B16] Wang JY Zhang HT Han JS Chang JY Woodward DJ Luo F Differential modulation of nociceptive neural responses in medial and lateral pain pathways by peripheral electrical stimulation: a multichannel recording study Brain Res 2004 1014 197 208 10.1016/j.brainres.2004.04.029 15213004

[B17] Yamamura H Iwata K Tsuboi Y Toda K Kitajima K Shimizu N Nomura H Hibiya J Fujita S Sumino R Morphological and electrophysiological properties of ACCx nociceptive neurons in rats Brain Res 1996 735 83 92 8905172 10.1016/0006-8993(96)00561-6

[B18] Sikes RW Vogt BA Nociceptive neurons in area 24 of rabbit cingulate cortex J Neurophysiol 1992 68 1720 1732 1479441 10.1152/jn.1992.68.5.1720

[B19] Xing GG Liu FY Qu XX Han JS Wan Y Long-term synaptic plasticity in the spinal dorsal horn and its modulation by electroacupuncture in rats with neuropathic pain Exp Neurol 2007 208 323 332 10.1016/j.expneurol.2007.09.004 17936754

[B20] Sun JJ Chuang Kung J Wang CC Chen SL Shyu BC Short-term facilitation in the anterior cingulate cortex following stimulation of the medial thalamus in the rat Brain Res 2006 1097 101 115 10.1016/j.brainres.2006.04.065 16725116

[B21] Gemmell C O'Mara SM Plasticity in the projection from the anterior thalamic nuclei to the anterior cingulate cortex in the rat *in vivo*: paired-pulse facilitation, long-term potentiation and short-term depression Neuroscience 2002 109 401 406 10.1016/S0306-4522(01)00554-1 11823054

[B22] Liu RJ Qiang M Qiao JT Nociceptive c-fos expression in supraspinal areas in avoidance of descending suppression at the spinal relay station Neuroscience 1998 85 1073 1087 10.1016/S0306-4522(97)00662-3 9681947

[B23] Wei F Zhuo M Potentiation of sensory responses in the anterior cingulate cortex following digit amputation in the anaesthetised rat J Physiol 2001 532 823 833 10.1111/j.1469-7793.2001.0823e.x 11313449 PMC2278568

